# *Gastrodia and Uncaria* (tianma gouteng) water extract exerts antioxidative and antiapoptotic effects against cerebral ischemia in vitro and in vivo

**DOI:** 10.1186/s13020-016-0097-6

**Published:** 2016-05-31

**Authors:** Jia Wen Xian, Angus Yiu-Ting Choi, Clara Bik-San Lau, Wing Nang Leung, Chun Fai Ng, Chun Wai Chan

**Affiliations:** School of Chinese Medicine, The Chinese University of Hong Kong, Shatin, New Territories, Hong Kong, People’s Republic of China; Institute of Chinese Medicine, The Chinese University of Hong Kong, Shatin, New Territories, Hong Kong, People’s Republic of China; State Key Laboratory of Phytochemistry and Plant Resources in West China, The Chinese University of Hong Kong, Shatin, New Territories, Hong Kong, People’s Republic of China

## Abstract

**Background:**

*Gastrodia and Uncaria* decoction (tianma gouteng yin) is commonly used in Chinese medicine to treat cerebral ischemia. The aim of this study was to investigate the neuroprotective effects of a water extract (GUW) of *Gastrodia elata* (tianma; GE) and *Uncaria rhynchophylla* (gouteng; UR) against ischemic insult using oxygen-glucose-deprived neuronal differentiated PC12 cells and rats subjected to middle cerebral artery occlusion (MCAO).

**Methods:**

GUW was prepared by boiling raw GE and UR in water, followed by the lyophilization of the resulting extract. Neuronal differentiated PC12 cells were subjected to oxygen-glucose deprivation with or without GUW. The neuroprotective effects of GUW were compared with those of the corresponding GE and UR extracts to tease apart the effects of the different herbs. The synergistic effect of GE and UR in GUW was measured using a modified version of Burgi’s formulae. The neuroprotective mechanisms via *Nrf2* and anti-apoptotic pathways were investigated using real time PCR and enzyme activity assays. The neuroprotective effects of GUW were studied in vivo using a rat MCAO model. Neurofunctional outcome and brain infarct volume we assessed. H&E staining, cresyl violet staining and immunohistochemistry were performed to assess the histological outcome.

**Results:**

The results of lactate dehydrogenase assay showed that GUW protected cells in a concentration-dependent manner (*P* < 0.001). Moreover, the neuroprotective effects of GUW were greater than those of GE + UR (*P* = 0.018). Burgi’s formula showed that the herbs in GUW acted synergistically to protect cells from ischemic injury. GUW significantly upregulated *Bcl*-*2* expression (*P* = 0.0130) and reduced caspase-3 activity by 60 % (*P* < 0.001). GUW upregulated *Nrf*-*2* expression (*P* = 0.0066) and the antioxidant response element pathway genes. The infarct volume was reduced by 55 % at day 7 of reperfusion (*P* < 0.001), and significant improvements were observed in the neurological deficit score and beam-walking test at 7 days (*P* < 0.001). H&E and cresyl violet staining revealed higher tissue integrity in the GUW treatment group compared with MCAO rats.

**Conclusion:**

GUW modulated the antioxidant system and antiapoptotic genes in oxygen-glucose deprived neuronal differentiated PC12 cells and MCAO sprague–dawley rats.

**Electronic supplementary material:**

The online version of this article (doi:10.1186/s13020-016-0097-6) contains supplementary material, which is available to authorized users.

## Background

Stroke is the third major cause of death in the world, with more than 80 % of strokes being caused by cerebral ischemia [1]. Recombinant plasminogen activator is currently the only FDA-approved drug for the treatment of acute stroke, but its effectiveness has been limited by its small therapeutic window and severe complications [2–4]. Moreover, the therapeutic dose of this drug is too low to affect the cellular mechanisms responsible for neuroprotection.

The reactive oxygen species (ROS) generated during ischemia are one of the major contributing factors to neuronal cell death [5]. Oxidative stress occurs when the rate at which ROS are cleared by the intracellular anti-oxidant system is slower than the rate at which they are produced. ROS can oxidize a variety of intracellular molecules such as DNA and lipids, eventually leading to apoptotic cell death [5]. Neurons are more susceptible to oxidative damage than most other cells because of their high metabolic rate and low expression levels of antioxidant enzymes [6].

The increase of antioxidant enzymes expression and activity might attenuate cellular ischemic injury. Nuclear factor erythroid 2-related factor 2 (*Nrf*-*2*) is a transcription factor that acts on the cis-acting antioxidant regulating element (ARE) to regulate the expression of antioxidant enzymes, including superoxide dismutase (SOD) and glutathione peroxidase (GPx) [7, 8]. The upregulation of *Nrf*-*2* and antioxidant enzymes effectively protected neurons from ischemic injury, improving histological outcomes and sensorimotor functioning of ischemic rats [9, 10]. Conversely, protein disulfide isomerase (PDI) activity prevents DNA fragmentation and cell death in hippocampal neurons from ischemic rats [11].

Chinese medicine has been used extensively to treat a variety of different brain diseases, and *Gastrodia Uncaria* decoction (tianma gouteng yin), in particular, is commonly used for treating cerebral ischemia and brain-related disease [12–16]. The main herbal components of *Gastrodia Uncaria* decoction include *Gastrodia elata* (*Tianma*; GE) and *Uncaria rhynchophylla* (*Gouteng*; UR). Several studies have demonstrated the prevention of ischemia-induced apoptosis by GE extracts and its active components [17–19]. Furthermore, the methanolic extracts of UR have been reported to exhibit neuroprotective effects against global cerebral ischemia in rats [20–22].

The aim of this study was to investigate the effects of the water extract of *Gastrodia elata* and *Uncaria rhynchophylla* (GUW) and its antioxidative mechanisms on oxygen-glucose deprived (OGD) neuronal differentiated PC12 cells and a rat middle cerebral artery occlusion (MCAO) model.

## Methods

### Materials

All the chemicals used in this study were purchased from Sigma-Aldrich (St. Louis, MO, USA) unless specified otherwise. Cell culture media and supplements were obtained from Life Technologies (Grand Island, NY, USA).

### Extraction methods

**Fig. 1:**
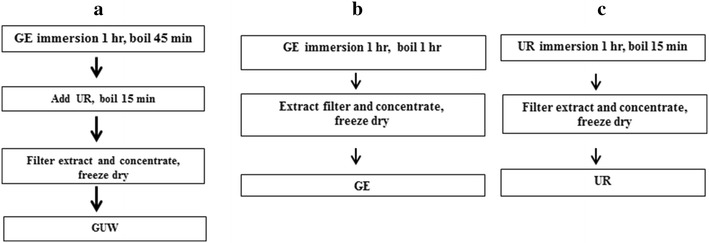
Extraction protocols of **a** *Gastrodia elata* and *Uncaria rhynchophylla* water extract (GUW), **b** *Gastrodia elata* water extract (GE) and **c** *Uncaria rhynchophylla* water extract (UR)

Crude GE and UR were purchased from Zhixin Medicine Health Co. (Guangdong, China). The herbal extracts were authenticated by Dr. Zheng Ling at the Institute of Chinese Medicine, The Chinese University of Hong Kong, based on the methods described in the 2010 edition of the Chinese Pharmacopoeia (Additional file 1) [23]. Voucher specimens of GE (Specimen No: 2010–3294) and UR (Specimen No: 2010–3295) were kept at the Institute of Chinese Medicine Museum, The Chinese University of Hong Kong. The GUW extract was prepared based on the boiling procedure used for preparing GE and UR in *Gastrodia and Uncaria* decoction [24]. Briefly, GE (107.1 g) was immersed in distilled water (2.5 L) for 1 h and boiled for 45 min. UR (142.9 g) was then added, and the resulting mixture was heated at reflux for 15 min. The mixture was then cooled to room temperature and filtered, and the resulting filtrate was rotary evaporated and lyophilized using a BÜCHI Rotavapor R-220 (Flawil, Switzerland) to give a powder (Fig. 1a).

Individual extracts GE and UR were prepared separately by immersing the individual herbs in water in a 1:10 (w/v) ratio for 1 h, and then heating the resulting mixtures at reflux for 1 h. The mixtures were then filtered to give the water-soluble fractions, and stored at room temperature for later use. This process was repeated for the herbal residues of GE and UR. Both fractions were pooled together to make the individual GE (Fig. 1b) and UR (Fig. 1c) water-soluble fractions. These water-soluble fractions were subsequently converted into powders using the methods described above.

### Cell line

PC12 cells (ATCC, Manassas, VA, USA) were grown in RPMI 1640 culture medium supplemented with 10 % horse serum, 5 % FBS and 100 U/mL penicillin G sodium. The cultures were maintained at 37 °C under 5 % CO_2_. The PC12 cells were pre-treated with nerve growth factor 7S (Millipore, Billerica, MA, USA) to obtain neuron-like cells [25].

### Cell viability measurements by MTT assay

Cell viability was assessed by using a 3-(4,5-dimethythiazol-2-yl)-2,5-diphenyl tetrazolium bromide (MTT) assay. Briefly, PC12 cells were seeded onto 96-well plates and incubated at 37 °C under 5 % CO_2_ for 24 h. The cells were treated with GE, UR or GUW extracts at concentrations in the range of 0–4000 μg/mL, and the resulting mixtures were incubated for 24 h. The cells were then treated with 0.5 mg/ml MTT and incubated at 37 °C under 5 % CO_2_ for 4 h. The medium was carefully aspirated and the remaining formazan crystals in each well were dissolved in dimethylsulfoxide (10 mL). The absorbance characteristics of the formazan solutions in each well were read at 490 nm using a μQuant plate reader (BioTek, Winooski, VT, USA). The relationship between the extract concentration and cell viability was visually determined. The pharmaceutical relationship between the GE and UR in GUW was calculated using a modified version of Burgi’s formula [26], as follow:

$$q = \frac{{E_{GE + UR} }}{{E_{GE} + E_{UR} - E_{GE} \times E_{UR} }}$$

E_x_: effectiveness of herbal extract x. For this formula, q < 0.85 indicates that the two drugs in question are acting in an antagonistic manner, whereas q > 1.15 indicates that the two drugs are acting in a synergistic manner. Values in between these limits (i.e., 0.85 < q < 1.15) indicate that the effects of the two herbs are additive.

### The effect of oxygen-glucose deprivation on PC12 cells

PC12 cells were seeded at a density of 1.5 × 10^4^ cells/well in poly-d-lysine-coated 6-well culture plates, and grown in RPMI 1640 culture medium supplemented with 10 % horse serum, 5 % FBS and 100 U/mL penicillin G sodium. After 24 h of incubation, neural differentiation was induced by the addition of a 50 ng/mL solution of nerve growth factor 7.0S (Millipore, Billerica, MA, USA) in RPMI 1640 culture medium containing 1 % horse serum and 0.5 % FBS. The cells were differentiated for 6 days and the differentiation medium was changed on alternate days. Oxygen-glucose deprivation (OGD) was applied on neural differentiated PC12 cells, as reported previously [27]. PC12 cells were cultured with glucose-free RPMI 1640 in an air-tight hypoxia chamber (STEMCELL Technologies Inc., Vancouver, Canada). Experimental groups were defined as follows: control, OGD, GUW, GE, UR and GE + UR. GUW was added to the different groups at concentrations of 1000, 2000 and 4000 μg/mL. Unless specified otherwise, the GUW treatment group was treated with 2000 μg/mL of GUW. The chamber was filled with anoxia gas composed of 95 % N_2_/5 % CO_2_ for 30 min at a flow rate of 3 L/min and the cells were then incubated for 8 h at 37 °C to simulate ischemia. For the control group, the cell cultures were subjected to the experimental procedures described above using normal RPMI 1640 as the culture medium and PBS as a vehicle, followed by incubation under normoxic conditions.

### Cytotoxicity assay

Cell death was measured after OGD based on the amount of lactate dehydrogenase (LDH) leaked into the culture medium using an LDH cytotoxicity detection kit (TaKaRa, Otsu, Japan), according to manufacturer’s instructions.

### Flow cytometry analysis of ROS

Intracellular ROS production was detected using a dichloro-dihydro-fluorescein diacetate (DCFH-DA) assay [28]. Briefly, 10 μM DCFH-DA was added to the PC12 cells after OGD, and the cells were then incubated at 37 °C for 30 min in the dark. Fluorescent signals were detected by flow cytometry using a FACSCanto™ II system (Becton–Dickinson, Franklin Lakes, NJ, USA), as reported previously [29]. The results were expressed as the relative percentage of DCF-fluorescence in the target groups compared with the cells in the control group.

### Real-time polymerase chain reaction (RT-PCR)

Total mRNA was prepared using the Trizol reagent (Invitrogen Life Technologies, Carlsbad, CA, USA) according to manufacturer’s instructions. cDNA was synthesized using the Promega Improm II Reverse Transcription System (Promega, Madison, Wi, USA) according to the manufacturer’s instructions. RT-PCR experiments were conducted at a final volume of 10 μL containing 5 μL of 2× SYBR^®^ Green I master mix (BIO-RAD, Hercules, CA, USA), 0.5 μL of forward and reverse primers, and 1.0 μL of cDNA diluted in 3 μL of DEPC H_2_O. The nucleotide sequences of the primers used in this experiment were as follows: (1) PDI, forward 5′-TCTGGAGGAGGAGGACAAC-3′, reverse 5′- TGGAAAACACATCGCTATT-3′; (2) *Bcl*-*2*, forward 5′-ATACCTGGGCCACAAGTGAG-3′, reverse 5′-TGATTTGACCATTTGCCTGA-3′; (3) *Nrf*-*2*, forward 5′-GAGACGGCCATGACTGAT-3′, reverse 5′-GTGAGGGGATCGATGAGTAA-3′; and (4) β-actin, forward 5′-GAGGCCCCTCTGAACCCTAA-3′, reverse 5′-ACCAGAGGCATACAGGGACAA-3′. A comparative CT method was used to compare the fold difference in the expression of these systems relative to the control.

### Superoxide dismutase (SOD) assay

The SOD activity in the PC12 cells after OGD was measured using a SOD assay kit (Cayman Chemical, Ann Arbor, MI, USA). Tetrazolium salt, xanthine oxidase and cell lysate were added to a 96-well plate. The OD540 value of the formazan produced was measured by a fluorescent microplate reader (FLUOstar OPTIMA, BMG LABTECH, Ortenberg, Germany). The results of these experiments were expressed as the relative percentage of SOD activity of each sample compared with the control.

### Glutathione peroxidase assay

The GPx levels in the PC12 cells after OGD were measured using a Glutathione Peroxidase assay kit (Cayman Chemical) according to manufacturer’s instructions. The activity of GPx was estimated by measuring the optical density at 340 nm (OD340) using a Quant system (BioTek).

### Catalase assay

The catalase activity of the PC12 cells was measured after OGD and the subsequent drug treatment processes using a catalase fluorometric detection kit (Enzo Life Sciences, Farmingdale, NY, USA), following the manufacturer’s instructions. The optical density at 595 nm (OD595) of resorufin was recorded. The results were expressed as the relative percentage of catalase activity of each sample compared with the control cells.

### Caspase-3 assay

After OGD, the PC12 cells were lysed with lysis buffer (Thermo Scientific, Waltham, MA, USA). Following two freeze–thaw cycles, the cell lysates were centrifuged at 21,500×*g* (Heraeus Multifuge 1S-R with Microliter Rotor, Thermo Scientific) for 3 min at 4 °C. The supernatants were subsequently collected and stored at –80 °C prior to being analyzed. The caspase-3 activity of the lysates was measured using a caspase-3 colorimetric protease assay kit (Thermo Scientific). The results were expressed as the relative percentage of caspase-3 activity of each sample compared with the control cells.

### Animals

Male Sprague–Dawley rats, weighing 260–280 g, were housed in cages (45 × 30 × 20 cm^3^) on 12-h light/dark cycle with ad libitum access to chow and water. The animal studies were performed following the ARRIVE guideline (Additional file 2). All of the experimental animal procedures used in this study were conducted with the approval of the Ethics Committee of the Chinese University of Hong Kong (AEEC ref number: 11/068/MIS-5) (Additional files 3, 4, 5).

### Middle cerebral artery occlusion (MCAO)

Rats were arbitrarily divided into three groups: GUW treatment, Control and Sham groups. After MCAO or a sham operation, the rats in the control and sham groups were orally administered with distilled water, whereas the rats in the GUW treatment group were administered with 288.6 mg/kg GUW once daily by oral gavage using an intragastric tube for 7 consecutive days.

Sprague–Dawley rats were intraperitoneally anesthetized with 400 mg/kg of chloral hydrate (VWR International Ltd, Poole, England). The cerebral blood flow (CBF) in these animals was intraoperatively monitored by laser Doppler flowmetery (PeriFlux System 5000, Perimed AB, Stockholm, Sweden). The probe holder (Probe holder PH07-6, Perimed AB) was located at the right cerebrum at 1 mm posterior and 2 mm lateral to the bregma above the skull. Briefly, the external carotid artery (ECA) and its branches were isolated and ligated. A round tip 4-0 nylon suture was inserted into the internal carotid artery (ICA) through the opening at the ECA and advanced to the anterior cerebral artery to occlude the middle cerebral artery (MCA). Once the MCA was occluded, the CBF was reduced by 60 %. After 2 h of occlusion-induced ischemia, reperfusion was allowed by the gentle removal of the nylon filament. For the sham rats, the left CCA and ECA were exposed without inserting the filament into the ICA.

### Infarct volume assessment

Brain infarct volume was quantified by 2,3,5-triphenyltetrazolium chloride (TTC) staining [30, 31]. Rats were euthanized with an overdose of pentobarbital at 7 days post-operation. The brains of the rats were harvested and cut into slices of 2 mm in thickness. The fresh brain slices were immersed in 2 % TTC solution in PBS for 20 min at 37 °C and their images were subsequently captured using an Epson Perfection 1260 desktop scanner (Seiko Epson Corporation, Nagano, Japan) at a resolution of 600 dpi. The area of the white infarct region was then analyzed using Adobe Photoshop CS4 Extended Version 11.0.1 (Adobe Systems Inc., San Jose, CA, USA). The infarct volume of a slice was calculated using the following equation:

$${\text{Infarct volume }} = {\text{Infarct area of the slice }} \times {\text{thickness of the slice }}(2\,{\text{mm}})$$

The total infarct volume was calculated by summing up the infarct volumes of the different slices.

### Cresyl violet staining

At the end of the experiment at day 7, the animals were anesthetized and their brains were perfused transcardially with phosphate buffered saline, followed by 4 % paraformaldehyde. The brains were subsequently excised, embedded with paraffin wax and cut coronally into slices of 6-µm thickness. These sections were then stained with 0.1 % cresyl violet for 10 min.

### Immunohistochemistry

The expression levels of *Nrf*-*2* and *Bcl*-*2* at the penumbra of the rats were examined by immunohistochemistry. At 7 days after MCAO, the rats were euthanized and their brains were harvested and cut into slices of 6-µm thickness, as above mentioned. These sections were then blocked with FBS containing 10 % goat serum and 1 % BSA, and incubated with rabbit polyclonal anti-*Nrf*-*2* (1:200; Abcam, Cambridge, UK) and rabbit polyclonal anti-*Bcl*-*2* (1:400; Abcam) overnight at 4 °C. The slices were then washed with PBS and incubated with Alexa Fluor^®^ 555 Goat Anti-Rabbit IgG (H + L) (Invitrogen Life Technologies) for 1 h with dilutions of 1:400 and 1:800 for binding on anti-*Nrf*-*2* and anti-*Bcl*-*2*, respectively. The slices were then washed with PBS and counterstained with DAPI. Fluorescence images were captured with ApoTome.2 system (Carl Zeiss AG, Jena, Germany).

### Neurological deficit score

Neurological deficit score (NDS) was performed using a modified Bederson’s scoring system to assess the diagnosis and prognosis of GUW treatment on cerebral ischemia [32]. NDS 1: Rats showing contralateral limb flexion (to the left limbs). NDS 2: Rats showing decreased resistance to lateral push to the left. NDS 3: Rats showing contralateral circling, *i.e.*, circling to the left only. NDS 4: Rats showing spontaneous circling. The NDSs were measured at 1, 2, 3, 5 and 7 days after the MCAO.

### Beam-walking test

A beam-walking test was applied to assess the motor coordination and balance control of the forelimbs and hind limbs of rats treated with the different extracts to evaluate their motor functional recovery after ischemia [33, 34]. The experimental animals were trained to complete a beam-walking test 3 days before undergoing MCAO to take baseline measurements, which involved them traversing an elevated narrow beam (2.5 × 120 cm, 80 cm above the floor) [35]. During the training days (2 days), the rats required less than 10 s to cross the beam. The rats were motivated to cross the beam by placing their home cage at the opposite end of the training stage. The rats were given 90 s to cross the walkway to their home cage. Performance was assessed by recording the mean latency of three trials to walk along the beam. This beam-walking test was performed at 1, 2, 3, 5 and 7 days after MCAO.

### Statistical analysis

All data have been presented as the mean values ± SD. Student’s *t* test and one way ANOVA, followed by Dunnett’s multiple comparison test (GraphPad Prism 5.0 software; San Diego, CA, USA), were used for the statistical analysis. Statistical significance was taken as *P* < 0.05.

## Results

### Cell viability measurements by MTT assay

**Fig. 2:**
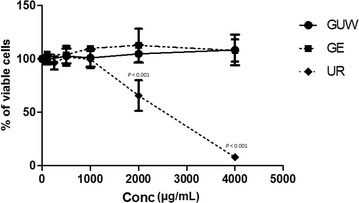
Cell response curve of various *Gastrodia elata* and *Uncaria rhynchophylla* extracts. The extracts of GUW and GE did not demonstrate toxicity to PC12 cells even at high concentration of 4000 µg/mL, whereas the UR extract showed toxicity to PC12 cells at concentrations higher than 1000 µg/mL vs control by one way ANOVA, post hoc Dunnett’s test (n = 4)

An MTT assay was used to determine the optimal concentration of each extract to be used on the PC12 cells. The GUW and GE extracts were found to be non-toxic to PC12 cells at concentrations below 4000 µg/mL (Fig. 2). However, the UR extract exhibited significant toxicity at concentrations greater than 2000 μg/mL (*P* < 0.001) and gave an IC_50_ value of 2557 μg/mL.

### Effect of GUW on the morphological characteristics of PC12 cells following OGD

**Fig. 3:**
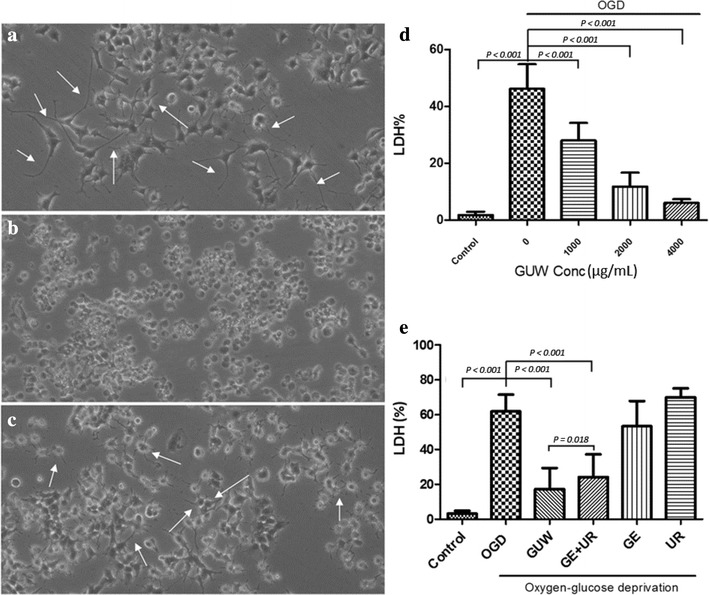
Morphologies of NGF-differentiated PC12 cells under OGD with or without GUW treatment. **a** NGF-treated PC12 cells differentiated with neurite extentions without OGD treatment. **b** After OGD, cell bodies shrank with a loss of neurite outgrowth. **c** PC12 cells retained neuronal morphology with GUW treatment. (Mag. 100×). *White arrows* indicate neurite protrusions. **d** PC12 cells injury/death was quantified by LDH assay after 8 h OGD. GUW showed concentration dependent protective effects on cells. **e** PC12 cells injury/death was quantified by measuring the release of LDH from injured cells. After OGD, single extracts of GE or UR only did not exhibit significant protective effect on OGD insult on NGF-treated PC12 cell. However, both GUW and the combination of GE and UR (GE + UR) water extracts have significant protective effects. Furthermore, GUW was also found to exert significantly greater neuroprotection better than GE + UR; vs control and vs OGD by one way ANOVA, post hoc Dunnett’s test (n = 4); vs GE + UR by Student’s *t* test (n = 4)

Neurite extensions were found in the nerve growth factor (NGF)-treated PC12 cells in the control group (Fig. 3a). In general, the cells in the OGD group become globular in shape with shorter neurite protrusions (Fig. 3b). GUW treatment did not lead to any discernible differences in the neuronal morphology and neurite branches of these cells (Fig. 3c).

### GUW extracts against OGD-induced cell injury

PC12 cells showed 70 % cell death after the OGD treatment process. The treatment of the cells with GUW provided them with significant protection from OGD-induced cell death (*P* < 0.001, Fig. 3d). Moreover, GUW exhibited neuroprotective effects in a concentration-dependent manner.

### Drug interaction of *Gastrodia elata* and *Uncaria rhynchophylla*

The individual GE and UR extracts (GE + UR) were mixed to form the same composition as that of GUW to investigate whether the protective effects of GUW were acting in an addictive or synergistic manner and study the pharmaceutical relationship between the two herbs in GUW. The GE- and UR-only treatments did not provide significant protection to the cells from the OGD insult (Fig. 3e). Both GUW (*P* < 0.001) and GE + UR (*P* < 0.001) significantly attenuated LDH release, while GUW exerted significantly greater neuroprotection than GE + UR (*P* = 0.018). The q value obtained in our study was 16, suggesting that the herbs in GUW were acting synergistically to protect the cells against OGD-induced cell death.

### Caspase-3 activity

**Fig. 4:**
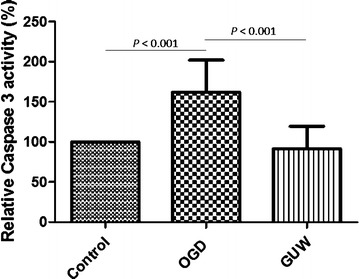
Changes in caspase-3 activity following OGD on NGF-treated PC12 cells. The activity of caspase-3 in the cell lysates were measured and normalized with the control group, vs OGD by one way ANOVA, post hoc Dunnett’s test (n = 4)

Caspase-3 activity increased significantly by 160 % (*P* < 0.001) compared with the control cells after exposure to OGD for 8 h (Fig. 4). However, the caspase-3 activity of the OGD groups returned to normal levels (*P* < 0.001) after they were treated with GUW (2000 μg/mL).

### mRNA expression after OGD

**Fig. 5:**
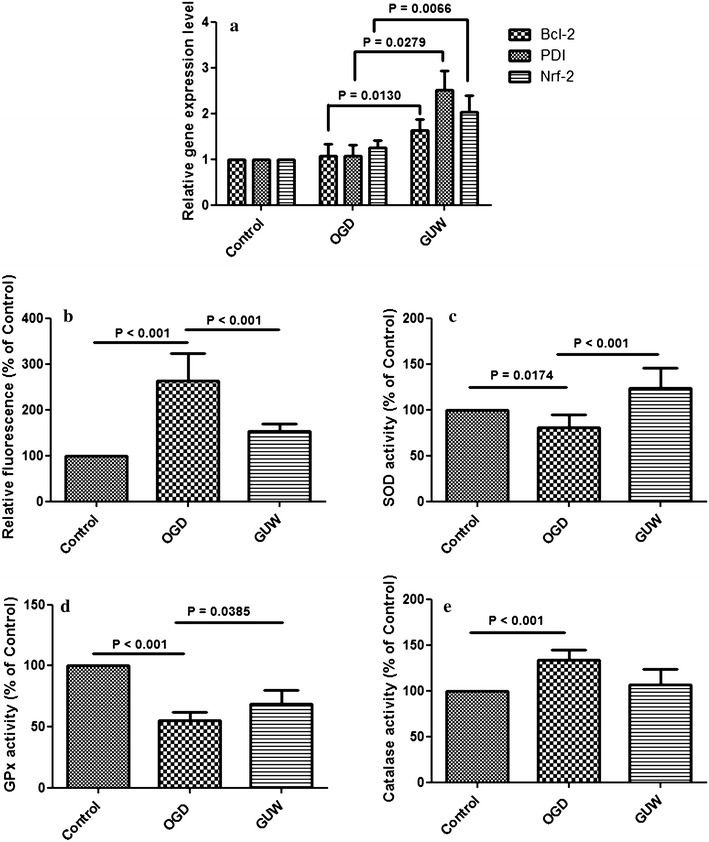
**a** mRNA expression levels of antioxidant and antiapoptotic genes after OGD and GUW treatment. Ct values of the genes of interest were first normalized to intrinsic β-actin of each sample, then the fold differences among each group and control was them calculated, vs OGD by one way ANOVA, post hoc Dunnett’s test (n = 4). Status of oxidative stress after OGD and GUW treatment. **b** Reactive oxygen species amount in between the control, OGD and GUW -treated group. The concentration of intracellular ROS was analyzed by flow cytometry. **c** SOD, **d** GPx and **e** Catalase activity in NGF-treated PC12 cells in different treatment groups, vs control and vs OGD by one way ANOVA, post hoc Dunnett’s test (n = 4)

The mRNA expression levels of *Bcl*-*2*, *PDI* and *Nrf*-*2* in the GUW group were 1.6-(*P* = 0.0130), 2.4-(*P* = 0.0279) and 1.5-fold (*P* = 0.0066) higher than those of the OGD group, whereas no significant changes were observed in these levels between the OGD and control groups (Fig. 5a). The expression of the housekeeping gene *β*-*actin* was measured to normalize the expression levels of the target genes.

### Flow cytometry analysis of reactive oxygen species

OGD led to a 2.5-fold increase in the quantity of intracellular ROS compared with the control group. However, we also observed a significant decrease of 40 % in the quantity of ROS in the GUW samples compared with the OGD group (*P* < 0.001; Fig. 5b).

### Activity of antioxidant enzymes

The SOD activity of the NGF-treated PC12 cells exposed to OGD was 80 % lower than that of the control group (*P* = 0.0174; Fig. 5c). The treatment of these cells with GUW led to a 40 % increase in SOD activity compared with the OGD group (*P* < 0.001). The GPx and catalase activities of the NGF-treated PC12 cells decreased following their exposure to OGD (*P* < 0.001), whereas the treatment of these cells with GUW effectively restored their GPx activity (*P* = 0.0385; Fig. 5d). The catalase activity of the NGF-treated PC12 cells increased significantly by 35 % in the OGD group compared with the control group (*P* < 0.001) (Fig. 5e), but no significant difference was found in the catalase activity between the OGD and GUW treatment groups.

### GUW reduced the infarct volume and improved the histological outcome after MCAO

**Fig. 6:**
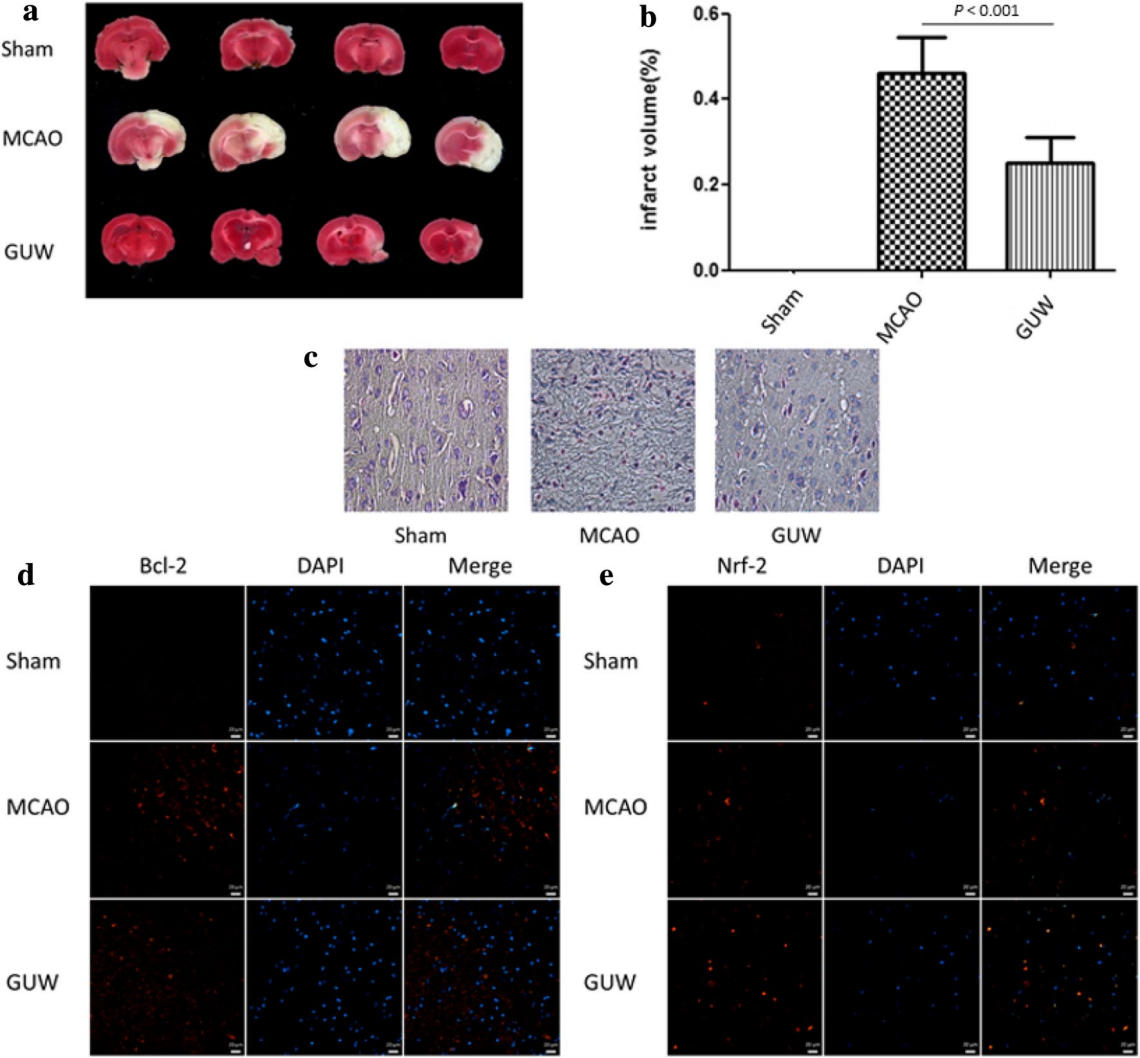
GUW reduced brain infarction volume and improved histological outcome. Brains were harvested 7 days of post-operation. The brains were sliced and performed TTC staining to evaluate the infarction volume and cresyl violet staining to examine the neuronal morphology at the infarct region. **a** The percentage of brain infarction of different groups of rats at 7 days of post-operation. **b** Data are expressed as mean ± SD, vs MCAO control group by one-way ANOVA (n = 10). **c** GUW increased the number of cells with normal neuronal morphology and decreased the number of shrunken and misshapen cells in cresyl violet–stained sections (magnification factor 400×). The *Bcl*-*2* and *Nrf*-*2* expression at the penumbra was examined by immunohistochemistry. GUW showed to upregulate both proteins in the penumbra. Representative images of the expression of **d** *Bcl*-*2* and **e** *Nrf*-*2*. *Scale bar *20 µm

The MCAO operation inflicted damage to different areas on the right hemisphere of the brain, including the cortex, hippocampus and basal ganglia cortex (Fig. 6a). GUW treatment led to a significant reduction in the infarct volume of brain by 55.7 % (*P* < 0.001) compared with the control group at 7 days post-operation (Fig. 6b).

Cresyl violet staining of the coronal brain sections taken from the rats in the MCAO group showed a dramatic decrease in the size of the Nissl bodies, as well as an increase in the size of the intracellular space between them (Fig. 6c). Notably, the administration of GUW rescued the neurons in the infarct region and preserved the tissue structure.

### GUW led to increased expression levels of *Bcl*-*2* and *Nrf*-*2*

Immunohistochemistry was used to examine the expression levels of *Bcl*-*2* and *Nrf*-*2* 7 days after MCAO. The results revealed increases in the expression levels of *Bcl*-*2* (Fig. 6d) and *Nrf*-*2* (Fig. 6e) at the penumbra of the MCAO group. Further increases in the expression levels of these two proteins were observed in the GUW treatment group, suggesting that GUW modulated the expression levels of *Bcl*-*2* and *Nrf*-*2* in vivo.

### GUW resulted in improved neurological function after MCAO

**Fig. 7:**
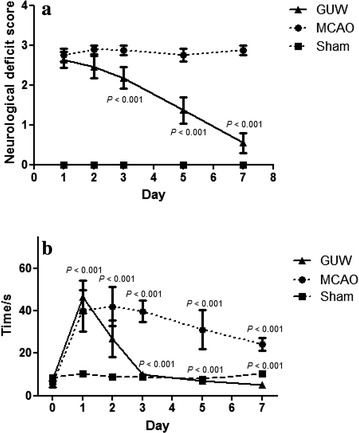
Sprague Dawley rats showed recovery of neurological and locomotor function after receiving GUW treatment. **a** Daily neurological deficit score measurement of different groups. Neurological deficit score was measured and recorded on day 1, 3, 5, 7 after MCAO surgery. MCAO control group received distilled water as vehicle. Data are expressed as mean ± SD, vs control group by one way ANOVA (n = 10). **b** Results of beam walking test after brain injury. After MCAO operation, rats receiving either vehicle or GUW showed significantly longer latencies to across the beam compared to sham-injured rats. At first 3 days treatment, the time to cross beam of GUW group was shorter time than the MCAO group significantly. Data are expressed as mean ± SD, vs sham group and vs MCAO group, by one way ANOVA (n = 10)

The NDS of the rats was measured on a daily basis after 2 h of ischemia and 24 h of reperfusion. The MCAO rats scored higher than 3. Notably, GUW treatment led to a lower score after day 3 compared with the MCAO control (*P* < 0.001; Fig. 7a).

### GUW led to improved behavior in the Beam-walking test after MCAO-injury

The time taken for rats to walk across the beam was recorded daily. The MCAO control rats took 31 ± 21 to 42 ± 33 s to walk along the wooden beam throughout the experimental period, which was fourfold higher than that of the sham group (*P* < 0.001). A significantly faster walking time was observed in the GUW-treated rats after 3 days of drug treatment. For example, the time taken to cross the beam in this group decreased from 39 ± 27 s at day 1 to 11 ± 6 s at day 3 (*P* < 0.001), resulting in performance comparable with that of the sham group rats (Fig. 7b).

## Discussion

In this study, we investigated the protective effects of GUW on NGF-treated PC12 cells subjected to OGD by measuring LDH release. GUW protected NGF-treated PC12 cells against OGD-induced cell death and exhibited dose-dependent neuroprotective effects. Moreover, the neuroprotection effects of GUW were greater than those of the individual GE and UR extracts. Flow cytometry results revealed that GUW led to a significant decrease in the amount of ROS, resulting in lower caspase-3 activity. Along with the increase observed in the regulation of *Bcl*-*2* by RT-PCR, these findings suggest that GUW could prevent cells from undergoing apoptosis as a consequence of ischemic insults.

Oxidative stress is one of the major contributors to the pathophysiological consequences observed in cerebral ischemia [36–38]. Oxidative stress also participates in excitotoxic neuronal necrosis, leading to systemic oxidative damage and apoptosis [36, 39]. To determine whether GUW could modulate the antioxidant protective mechanism in cells, we studied its effect on the activities of several antioxidant enzymes. *Nrf*-*2* is a basic leucine zipper redox-sensitive transcriptional factor that regulates the expression of several cellular antioxidant and cytoprotective genes from the ARE [7, 40, 41]. The upregulation of *Nrf*-*2* has been reported to mitigate oxidative stress-induced tissue injury in vivo [10, 42]. In contrast, the overexpression of PDI may lead to increased resistance to hypoxic or ischemic injury in vitro and in vivo [43]. Moreover, PDI enhances chaperone activity to protect against oxidative stress injury, suggesting that PDI may participate in more than one mechanism to protect neuronal cells from ischemic injury [43, 44]. Cerebral ischemia led to decreases in GPx and SOD activity, as well as an increase in catalase activity [45]. GUW rescued the expression of GPx and SOD activity, and had no discernible impact on the catalase activity. GUW could therefore modulate the *Nrf*-*2*-ARE pathway and enhance the antioxidant mechanism to protect the cells from ROS-induced oxidative damage.

Although most drugs show promising in vitro results, their application in vivo has been limited by their poor blood–brain barrier permeability [46]. The active components in GUW could penetrate the blood–brain barrier in vivo [47–49]. The in vivo efficacy of GUW was investigated using a MCAO model. Although it is not possible to rescue the neurons in the infarction core, the neurons in the penumbra region may be salvaged from ischemic/reperfusion injury [50]. The results of our study showed a smaller infarct area after GUW treatment compared with the control animals, suggesting that GUW rescued the cells in the penumbra. The immunohistochemistry results showed an upregulation in the levels of *Bcl*-*2* and *Nrf*-*2* at the penumbra in the GUW treatment group. This result was consistent with the in vitro results, which showed that GUW modulated the antiapoptotic and antioxidative genes to protect the brain from cerebral ischemia. Notably, the treatment of the MCAO rats with GUW led to a reduction in their NDS, as well as a decrease in the time required to complete the beam-walking test.

The results of our interaction study for GE and UR showed that GUW was a stronger neuroprotective agent than the GE + UR mixture, suggesting that GE and UR exhibit synergistic effects in GUW capable of supporting the survival of NGF-treated PC12 cells under OGD. It is therefore likely that the decocting process for the production of GUW resulted in the formation of the active components responsible for the greater neuroprotective effect [51, 52]. This study therefore represents the first report to show that the combination of GE and UR might lead to synergistic effects. Further chemical analysis is needed in the future to determine the root cause of this synergistic effect.

## Conclusion

GUW modulated the antioxidant system and antiapoptotic genes in neuronal differentiated PC12 cells at 2000 g/mL following oxygen-glucose deprivation and Sprague–Dawley rats at 288.6 mg/kg following middle cerebral artery occlusion.

## Additional files

10.1186/s13020-016-0097-6 Herbal authentication of *Gastrodia elata* and *Uncaria rhynchophylla*.
10.1186/s13020-016-0097-6 The ARRIVE guidelines checklist.
10.1186/s13020-016-0097-6 Animal experimentation ethics committee approval protocol 1.
10.1186/s13020-016-0097-6 Animal experimentation ethics committee approval protocol 2.
10.1186/s13020-016-0097-6 Animal experimentation ethics committee approval protocol 3.

## Abbreviations

ARE: antioxidant response element
DCFH-DA: dichloro-dihydro-fluorescein diacetate
GE: *Gastrodia elata*
GPx: glutathione peroxidase
GUW: *Gastrodia elata* and *Uncaria rhynchophylla* water extract
LDH: lactate dehydrogenase
MCAO: middle cerebral artery occlusion
MTT: 3-[4,5-dimethylthiazol-2-yl]-2,5-diphenyltetrazolium bromide
NGF: nerve growth factor
*Nrf-2*: nuclear factor ethyroid 2-related factor 2
OGD: oxygen-glucose deprivation
*PDI*: protein disulfide isomerase
ROS: reactive oxygen species
SOD: superoxide dismutase
TTC: 2,3,5-triphenyltetrazolium chloride
UR: *Uncaria rhynchophylla*
