# Supplementary material for: Gastrodia and Uncaria (tianma gouteng) water extract exerts antioxidative and antiapoptotic effects against cerebral ischemia in vitro and in vivo
Source: Chin Med. 2016 May 31;11:27. doi: 10.1186/s13020-016-0097-6 (PMC4888490; doi:10.1186/s13020-016-0097-6)
Supplement: Supplementary file 1 — 10.1186/s13020-016-0097-6 Herbal authentication of Gastrodia elata and Uncaria rhynchophylla. [file 13020_2016_97_MOESM1_ESM.pdf]

Herbel Authentication

Thin layer chromatography (TLC) of two herbs and the HPLC of *Gastrodia elata* was carried out for the authentication as listed in Chinese Pharmacopoeia 2010 with slight modifications.

Gastrodia elata

The test herb (1g) and *Gastrodia elata* reference herb (1g) was powdered by crusher and was weighed to a 50 ml conical bottom flask. To the flask, 10 ml methanol was added and sonicated for 15 mins. The mixture was filtered with Whatman No. 1 (185mm in diameter) filter paper. The filtrate was evaporated under reduced pressure until drying up. The filtrate powder was re-dissolved with 50µl methanol by sonication for 1min as test solution. Gastrodin (5g) which acted as chemical marker, was dissolved with methanol to make final concentration of 1g/ml. The *Gastrodia elata* reference herb, sample *Gastrodia elata* herb, test *Gastrodia elata* herb and chemical marker of gastrodin solutions were applied onto a TLC plate in separate bands (Silica gel 60 F_{254}, Merck KGaA, Darmstadt, Germany). The plate was put in a TLC tank pre-saturated with the developing solution (ethyl acetate: methanol: water = 7:3:1). Finally, the TLC plate was dried up and observed under visible light. Fig. 1 shows the details of the TLC profile of Gastrodia elata in developing system.

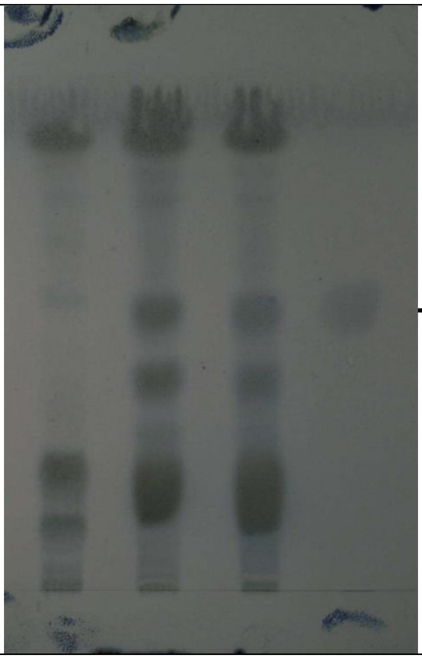

1<br>GE reference Herb	2<br>Reference herb GE from Zhixin	3<br>GE test sample purchased from Zhixin	4<br>Gastrodin
**Plate:** silica gel 60 F254 (Merck, Germany)			
**Developing system:** Ethyl acetate: Methanol: Water = 7:3:1			
**Detection:** visible light			
**Staining method:** 5 % phosphomolybdic acid in 50 % ethanol, heated.			

Figure 1. Authentication of *Gastrodia elata* by TLC chromatograms. TLC chromatograms of gastrodin standard and *Gastrodia elata* (GE) developed in ethyl acetate: methanol: water (7:3:1) solvent system. The Rt value of gastrodin in this developing system is 0.6. TLC was carried out according to the Chinese Pharmacopoeia 2010 with reference herb.

For HPLC analysis of *Gastrodia elata*, the mobile phase included a gradient elution of acetonitrile (ACN) and water. The linear gradient was 5% to 40% ACN in 60 min. The flow rate was set at 1 ml/min. The detector was operated at 220 nm and the column temperature was maintained at 25 °C. The concentration of gastrodin, 4-hydroxybenzyl alcohol (HBA) in the extracts were assessed.

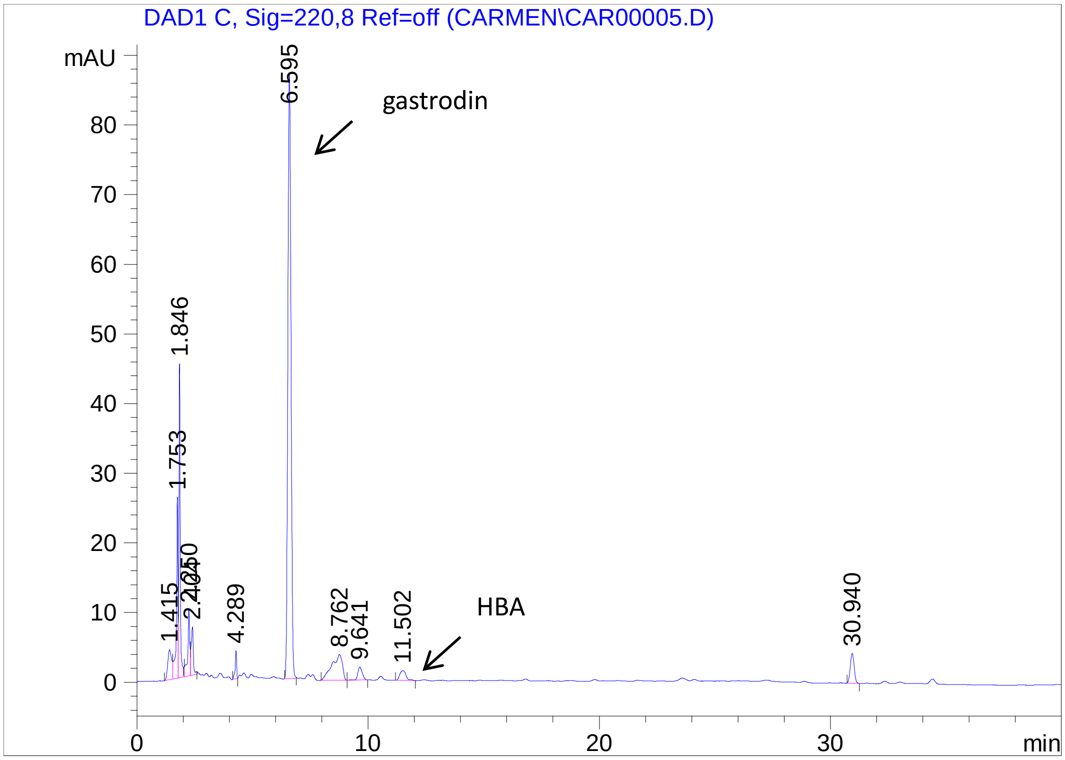

Figure 2. The chromatograms profile of *Gastrodia ealta*. The amount of Gastrodin and 4-hydroxybenzyl alcohol in *Gastrodia ealta* are 17.59±0.152 and 1.78±0.103 mg/g respectively.

Uncaria rhynchophylla

The test herb (3g) and *Uncaria rhynchophylla* reference herb (3g) was powdered by crusher and was transferred to a 50 ml round bottom flask with 15 ml of ammonia solution for 1 hour at room temperature. It was then extracted with ethyl acetate for two times (20 ml each time) by sonication for 10 min and filtered with Whatman No. 1 filter paper (185mm in diameter). The filtrate was dried up under low pressure. The filtrate powder was re-dissolved with methanol as the test solution. The *Uncaria rhynchophylla* reference herb, sample *Uncaria rhynchophylla* herb, test *Uncaria rhynchophylla* herb solution were applied as separate bands onto a TLC plate (Silica gel 60 F_{254}, Merck KGaA, Darmstadt, Germany). The plate was put in a TLC tank pre-saturated with the developing solution (Chloroform: ethyl acetate = 21:4, 30% ammonia solution saturation, developing system 1). The TLC plate was dried and observed under UV light at 254nm and 365nm, and after Sprayed with Dragendorff reagent, observed under viable light. Figure 3 shows the details of the TLC profile of *Uncaria rhynchophylla* .

<table>
<tr><td>UV254</td><td>UV365</td><td>UV-Vis (Sprayed with Dragendorff reagent)</td></tr>
<tr><td>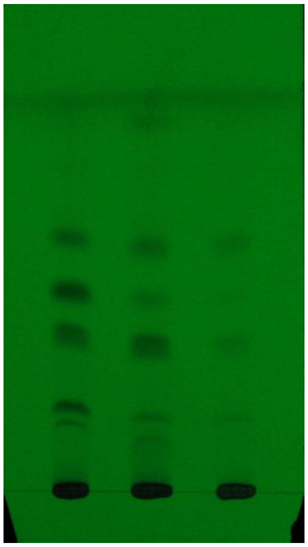</td><td>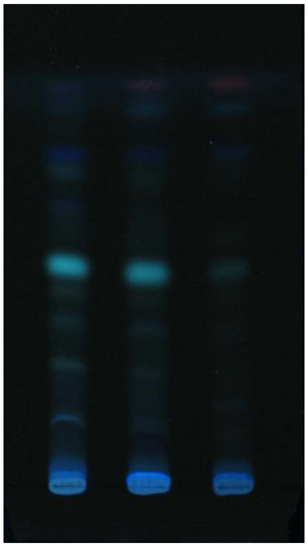</td><td>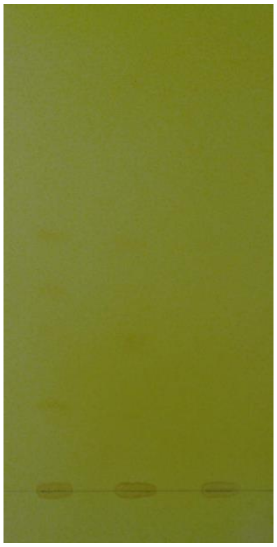</td></tr>
<tr><td>1 2 3</td><td>1 2 3</td><td>1 2 3</td></tr>
<tr><td colspan="3">1: UR reference Herb 2: Sample herb UR from Zhixin 3: UR purchased from Zhixin</td></tr>
<tr><td colspan="3">Plate: silica gel 60 F254 (Merck, Germany)</td></tr>
<tr><td colspan="3">Developing system: Chloroform: ethyl acetate = 21:4, 30 % ammonia solution aturation</td></tr>
<tr><td colspan="3">Detection: UV light at 254 nm and 365 nm, Dragendorff reagent→Visible light</td></tr>
<tr><td colspan="3">Staining method: Dragendorff reagent</td></tr>
</table>

Figure 3. Authentication of Uncaria Ramulus by TLC chromatograms. TLC chromatograms of *Uncaria rhynchophylla* (UR) developed in chloroform: ethyl acetate (21:4) solvent system. TLC was carried out according to the Chinese Pharmacopoeia 2010 with reference herb.

Reference:

1. Chinese Pharmacopoeia Commission. Pharmacopoeia of the People's Republic of China 2010 – book 1. Beijing: China Medical Science Press; 2010.
