# Supplementary material for: Gastrodia and Uncaria (tianma gouteng) water extract exerts antioxidative and antiapoptotic effects against cerebral ischemia in vitro and in vivo
Source: Chin Med. 2016 May 31;11:27. doi: 10.1186/s13020-016-0097-6 (PMC4888490; doi:10.1186/s13020-016-0097-6)
Supplement: Supplementary file 4 — 10.1186/s13020-016-0097-6 Animal experimentation ethics committee approval protocol 2. [file 13020_2016_97_MOESM4_ESM.pdf]

11/068/MIS

Animal Experimentation Ethics Committee (AEEC)

Application Form For Research

Proposal for use of experimental animals for research purposes at The Chinese University of Hong Kong

	Official Use

Project Title:

The neuro-protective effect of Tianma-Gouteng formula on cerebral ischemia

1. Summary of proposal

A description of the rationale, objectives and methodology of the proposal (copy of full grant proposal is not acceptable).

The objectives of this proposal is to explore the efficacy of a Chinese medicine formula composed of water extract of Tianma (Rhizoma Gastrodiae Elatae) and Gouteng (Ramulus Uncariae cum Uncis) (as TGW) in preventing brain damage and in promoting neuro-regeneration. The present research will provide a platform for studying the biological mechanisms of action of the formula and efficacy by animal model, with detailed authentication of the herbs involved .

All SD rat (young adult, male) will be subjected to middle cerebral artery occlusion (MCAO) surgery.The animals will be under general anaesthesia with mixture of ketamine and xylazine. The left common carotid artery (CCA) will be exposed and the external carotid artery (ECA) and its branches will be isolated and ligated. A 4-0 nylon suture with a round tip will be inserted into the internal carotid artery (ICA) through the opening at external carotid artery and advanced to the anterior cerebral artery to occlude the middle carotid artery (MCA). Once the MCA is blocked, the cerebral blood flow (CBF) signal which is monited by laser doppler flowmetry, will drop by about 60%. After 120 min blockage of MCA leading to cerebral ischemia, brain is reperfused with blood by removal of nylon filament gently. After 24h reperfusion , the neurological defect score is performed by animal behaviour.

The MCAO model rats will be randomly divided into three treatment groups (Low-, Mid- and High-dosage of TGW), negative (water) and positive control (Tempol) and sham groups.

After 7 days oral adminstration of TGW extract or control groups, MCAO operated rats are subject to four assessments in each group. One sub-group of animal will be subjected to cognitive assessment by shuttle box escape test. Other three sub-groups of animal will be aspirated cerebrospinal fluid for analysis its containing TGW ingredients, for brain tissue enzyme assessment and the evaluation of brain.

2. Academic and other staff involved in the experimental procedures (see Useful Information, item 2 and 6)

(a) Animal Licence

	Name	Licence No.
PI	LEUNG, Wing Nang Albert	(11-11) in DH/HA&P/8/2/1 Pt.16
CoI	CHAN, Chun Wai	(10-556) in DH/HA&P/8/2/1 Pt.16
CoI	XIAN, Jia Wen	(10-550) in DH/HA&P/ 8/2/1 Pt.16

*****Please attach a copy of current animal licences for above workers*****

(b) Brief description of experience of staff named in 2(a) with regard to animal experimentation.

Prof. Albert LEUNG has been working on projects using animals more than 15 years.
Prof. CW CHAN has been working on projects using animals more than 15 years.
Ms JW XIAN has been working on projects using animal more than 6 years.

3. Advancement of scientific / medical knowledge and the implementation of 3R's (Replace, Reduce, Refine) into the project

(a) A clear explanation of how this project advances scientific / medical knowledge.

The project use evidence based approach to investigate the effect and mechanism how tinama-guoteng fomula treat cerebral ischemia. The development of tianma-guoteng formula will shed light on modernisation of Chinese medicine on stroke treatment.

(b) Please describe where efforts have been made to **Replace** animals with "lower order of animals" alternatives, **Reduce** the number of animals used, and **Refine** the protocols to minimize suffering (See Useful Information, item 5).

Due to the heterogeneity and complexity of stroke patients, no alternatives other than experiments on live animals can be considered and processed.
Before performing animal model, we have applied the TGW extracts on the cell culture to find the effective dose on neuron cell as the reference dosing to apply animal model. Thus the number of animal is reduced.
Due to different assessments, the animals will also be euthanized immediately after the end of assessment or when the scientific endpoint is achieved in order to reduce any suffering of animals.

4. Types of experiments to be performed

Indicate the types of experiments that you propose to perform on the appropriate form. Use a separate form for each experiment. *(Please click the appropriate box)*

Short-term	Form 1	Euthanasia only	☐
	Form 2	Experiments performed wholly under anaesthesia followed by termination of the animals	☐
	Form 3	Experiments on conscious animals followed by termination of the animals	☐
Long-term	Form 4	Experiments on conscious animals	☐
	Form 5	Experiments on conscious animals with period(s) of anaesthesia	☒

* The definition of short-term experimentation is that the experimental procedures are to be finished within 24 hours. There is no holding of animals after 24 hours.

5. Hazards to personnel

NOTE: Applicant must complete the form "Health Safety and Environmental Assessment for Research Grant Applications" and submit details of the project for a full safety evaluation by the University Safety and Environment Office (USEO).

For the purpose of this ethics application (see Useful Information, item 9):

(a) Please provide a brief indication of potential serious hazards posed by any of the treatments specified in this application. Please include examples such as accidental injection, bite or scratch, chemical exposure, animal excretions that could potentially cause personnel hazard.

bite by animal

(b) Please describe details and safety measures to protect animal colonies, personnel and property from exposure if this project involves injection / administration of :

☐ cell lines / plasmids
☐ carcinogens
☐ cytotoxic agents
☐ microbiological organisms
☐ radiological hazards
☐ Other hazardous agents: _____

(*please click all that apply*)

N/A

6. Declaration

I have read and agree to abide by the Animal (Control of Experiments) Ordinance Cap 340 and the AEEC Guidelines.

Investigator Names	Signature	Date
LEUNG, Wing Nang Albert	[signature]	2011-9-22
CHAN, Chun Wai	[signature]	2011-9-22
XIAN, Jia Wen	XIAN Jia wen	2011-9-22

7. Acknowledgement by Department Chairman / School Director

Name	Signature	Department / School	Date
LEUNG, Wing Nang Albert	[signature]	School of Chinese Medicine	2011-9-22

	Official Use

LONG-TERM EXPERIMENTATION

FORM 5

Experiments on conscious animals involving period(s) of anaesthesia.
Full details of dosing and tissue (e.g. blood) collection regimens must be given.

A.	Brief description of experimentation	1.apply middle cerebral artery occlusion (MACO) to rats,and ischemia for 2h, and then reperfusion for 24h;<br>2.administer Tianma-Gouteng water extracts to MCAO rats for 7 days;<br>3.one groups for shuttle box escape test ;<br>4.one group for enzyme assessment;<br>5.one group for cerebrospinal fluid analysis;<br>6.one group for anlalysis the infarct volume.
B.	Animals<br>i. Species<br>ii. Sex<br>iii. Age<br>iv. Special characteristics<br>v. Total number<br><br>vi. Justification<br>(e.g. providing group size, number of groups, number of experiments etc.)	 SD rats<br>male<br>8 week(250g-280g)<br>N/A<br>360<br><br>There are 4 assesements in the experiment. For each assessment, 6 groups of rats will be applied, including 3 dosages of TGW extract (Low-, Mid- and High-dosage of TGW), negative (water) and positive control (Tempol) and sham groups.<br><br>Each group composes of 15 animals and, hence 15×6×4 = 360 rats are needed. Therefore, 360 rats are the minimum no. of rats necessary for the experiment.
C.	Location<br><br>i. Prior to experimentation Bldg<br>Room<br>ii. During experimentation Bldg<br>Room	 <br>LASEC<br>Breeding Area<br>LASEC<br>4/F
D.	Procedures	

i. Please describe the experimental procedures to be performed, list as 1, 2, 3 etc.<br><br>*Do not include those performed after death.*	1. The animals will be subjected to middle cerebral artery occlusion (MCAO). After the beginning of measuring cerebral blood flow (CBF), the rats will be under general anaesthesia with mixture of Ketamine and Xylazine (i.p. Ketamine 75mg/kg and Xylazine 5mg/kg), the left common carotid artery (CCA) will be exposed and the external carotid artery (ECA) and its branches were isolated and ligated. A 4-0 nylon suture will be inserted into the internal carotid artery (ICA) through the opening at ECA and advanced to the anterior cerebral artery to occlude the MCA. After 120 min ischemia, reperfusion will be started by removing the suture gently and the ECA will be permanently closed by cauterization. Afterwards, both incisions on scalp and neck will be closed.<br><br>2. According to the 4 assessments, the MCAO model rats will be divided into three treatment groups (Low-, Mid- and High-dosage of TGW), negative (water) and positive control (Tempol) and sham groups.<br><br>3. After 24 hours of middle cerebral artery occlusion, rats will be orally administered with extract of Tianma-Gouteng formula once daily until end of experiment.<br><br>4. Behavioral evaluation (Shuttle box escape test):<br>After 7 days oral administration of TGW extract or control groups, animals will be subjected to cognitive assessment by shuttle box escape test. In brief, single rat will be placed in the shuttle box and allowed to habituate to the environment for 300s and then will be subjected to 50 shuttle trials with fixed interval of 30s. In each trial, a tone signal (92dB) and the light signal will be presented for a maximum of 10s duration; rat will be allowed to avoid the electric shock by escaping to the other side of the shuttle box. If no conditioned response is occurred within 10s, a scrambled electric shock (0.9mA) will be delivered through the static grids for a maximum of 10s; rat will be allowed to escape from electric shock by moving to the other side of the shuttle box. If no unconditioned response occurred within 10s, electric shock will terminated automatically. The responses of the rats will be recorded by Shutavoid software automatically.<br>On day 7, 8, 9 (training series), and on day 13, 14, 15 (test series), the rats will be evaluated for their escape behavior in automated two-way shuttle-boxes. On day 15 after experiment, rats will be terminated immediately.<br><br>5. Enzyme assessment:<br>After 7 days oral administration of TGW extract or control groups, MCAO rats will terminated for brain tissue enzyme assessment .<br><br>6. Quantification of Brain Infarct Volume:<br>After 7 days oral administration of TGW extract or control groups, MCAO rats will terminated for evaluation of brain infarct volume.<br><br>7. Cerebral spinal fluid analysis:<br>After 7 days oral administration of TGW extract or control groups, MCAO rats will be aspirated cerebrospinal fluid for analysis its containing TGW ingredients. And rats will be anaesthetized during the experiment and terminated immediately after experiment.

	ii. Measurable Endpoints:<br>(i.e. please *click* the Scientific Endpoints that are to be achieved.)<br><br>Please *click* all that apply.	☒ body weight change<br>☐ tissue or blood samples<br>☐ change in the extent of tumour growth<br>☒ physiological data<br>☐ change in death rate or improved survival rate<br>☒ behavioural modification<br>☐ others, please specify:
E.	Details of anaesthesia<br>i. Agent<br>ii. Route of administration<br>iii. Dosage<br>iv. Monitoring of level of anaesthesia<br>v. frequency and duration of anaesthesia an individual animal would experience<br>*(please also comment on how many days recovery between each anaesthetic)*	Ketamine Xylazine<br>i.p<br>75mg/kg 5mg/kg<br>Depth of breathing, body gesture, no blinking eye<br>1. All rats will be anaesthetized for MCAO surgery and will last for 2 h. The animals will recover after the surgery.<br><br>2. 7 days after MCAO, only the sub-groups of Cerebrbral Spinal Fluid assessment will receive the second anaesthesia and the duration is 1 h (terminal).
F.	Details of analgesia during surgery<br>i. agent<br>ii. route of administration<br>iii. dosage	Buprenorphine HCl<br>i.p.<br>0.02mg/kg
G.	Details of analgesia following surgery or for other purposes<br>i. agent<br>ii. route of administration<br>iii. dosage	Buprenorphine HCl<br>i.p.<br>0.02mg/kg/day
H.	Animal Welfare<br>i. How often will the animals be monitored for their well-being ?<br><br>ii. Person(s) who will monitor well-being of animals<br>*(please give the name and post of staff / student and contact phone no.)*<br><br>iii. Will the procedure/surgery limit the ability of the animal move freely, feed and groom itself?	each day after MCAO surgery<br><br>XIAN Jia Wen PhD. student 26961231<br><br>☐ NO<br>☒ YES – Please provide justification and actions to minimize suffering: <u>this animal model is to mimic stroke , therefore, the animals will not be able to move very freely, but feed and groom is normal.</u>
I.	Humane Endpoints<br><br>If animals are found to be suffering during the course of this experiment, under what conditions the animals will be terminated?<br><br>Please *click* all that apply.	☒ ≥ 20% loss of body weight<br>☒ obvious distress<br>☐ extent of tumour growth - please give the size of tumour: _____<br>☐ others, please specify: _____
J.	Termination – clearly describe method to be used.	Intraperitoneal injection of over-dosage of 20% pentobarbital solution, about 0.2 ml.
K.	Justification if non-standard euthanasia method used	N/A

L.	Disposal method for carcasses, tissues and contaminated materials	The carcasses will be soaked with disinfectant for 30 minutes before disposal in the freezer of LASEC
M.	Disposal method for live animals	N/A

Form 2

Licence to Conduct Experiments

Name : LEUNG Wing Nang Albert [Ref No.: (11-11) in DH/HA&P/8/2/1 Pt.16]

Address : School of Chinese Medicine, The Chinese University of Hong Kong

By virtue of section 7 of the Animals (Control of Experiments) Ordinance, Chapter 340, the above-named is hereby licensed to conduct the type of experiment(s), at the place(s) and upon the conditions, hereinafter mentioned.

Type of experiment(s)

Rats will be used in the experiment. The animals will be subject to bilateral carotid artery occlusion (TIA) or middle cerebral artery occlusion (MCAO) under anaesthesia. For TIA, a midline incision will be made at the thoracic area. Both left and right common carotid arteries will be exposed and clamped for 12 minutes. For MCAO, a midline incision will be made at the thoracic area, the right internal carotid artery and external carotid artery will be exposed. An intraluminal suture will be inserted from an incision at the external carotid artery through the internal carotid artery to block the origin of the middle carotid artery transiently for 2 hours. The wound will then be closed. Analgesic agent will be given for pain relief. The animals will be divided into groups. Some animals will be fed with Chinese medicine formula/herbs and some animals will be fed with distilled water as control. The behavioural performances of MCAO-operated animals will be monitored by shuttle-box escape test. At the end of the experiment, cerebro-spinal fluid will be aspirated under anaesthesia for pharmacological studies. The animals will then be sacrificed by an overdose of anaesthetic, and tissues will be collected for further analysis. During the experiment, conditions of the animals will be monitored. Animals with serious injury will be euthanized before the end of the study.

Place(s) where experiment(s) may be conducted

Laboratory Animal Services Centre, The Chinese University of Hong Kong

---

Conditions

1. Such experiment(s) may only be conducted for the following purposes-

   To investigate the neuroprotective effects of one traditional Chinese medicine composed of Rhizoma Gastrodiae Elatae, Ramulus Uncariae cum Uncis, Radix Gentianae macrophyllae on TIA and MCAO rat model.

2. This licence is valid from 19 January 2011 to 18 January 2013

Dated 19 January 2011

DEPARTMENT OF HEALTH 衞生署 HONG KONG

Licensing Authority

Form 2

Licence to Conduct Experiments

Name : CHAN Chun Wai [Ref No.: (10-556) in DH/HA&P/8/2/1 Pt.16]

Address : School of Chinese Medicine, The Chinese University of Hong Kong

By virtue of section 7 of the Animals (Control of Experiments) Ordinance, Chapter 340, the above-named is hereby licensed to conduct the type of experiment(s), at the place(s) and upon the conditions, hereinafter mentioned.

Type of experiment(s)

Rats will be used in the experiment. The animals will be subject to bilateral carotid artery occlusion (TIA) or middle cerebral artery occlusion (MCAO) under anaesthesia. For TIA, a midline incision will be made at the thoracic area. Both left and right common carotid arteries will be exposed and clamped for 12 minutes. For MCAO, a midline incision will be made at the thoracic area, the right internal carotid artery and external carotid artery will be exposed. An intraluminal suture will be inserted from an incision at the external carotid artery through the internal carotid artery to block the origin of the middle carotid artery transiently for 2 hours. The wound will then be closed. Analgesic agent will be given for pain relief. The animals will be divided into groups. Some animals will be fed with Chinese medicine formula/herbs and some animals will be fed with distilled water as control. The behavioural performances of MCAO-operated animals will be monitored by shuttle-box escape test. At the end of the experiment, cerebro-spinal fluid will be aspirated under anaesthesia for pharmacological studies. The animals will then be sacrificed by an overdose of anaesthetic, and tissues will be collected for further analysis. During the experiment, conditions of the animals will be monitored. Animals with serious injury will be euthanized before the end of the study.

Place(s) where experiment(s) may be conducted

Laboratory Animal Services Centre, The Chinese University of Hong Kong

Conditions

1. Such experiment(s) may only be conducted for the following purposes-

To investigate the neuroprotective effects of one traditional Chinese medicines composed of Tianma (Rhizoma Gastrodiae Elatae) and Gouteng (Ramulus Uncariae cum Uncis) on TIA and MCAO rat model.

2. This licence is valid from 6 January 2011 to 5 January 2013

Dated 6 January 2011

DEPARTMENT OF HEALTH 衞生署 HONG KONG

Licensing Authority

Form 2

Licence to Conduct Experiments

Name : XIAN Jia Wen [Ref No.: (10-550) in DH/HA&P/8/2/1 Pt.16]
Address : School of Chinese Medicine, The Chinese University of Hong Kong

By virtue of section 7 of the Animals (Control of Experiments) Ordinance, Chapter 340, the above-named is hereby licensed to conduct the type of experiment(s), at the place(s) and upon the conditions, hereinafter mentioned.

Type of experiment(s)

Rats will be used in the experiment. Stroke will be induced in the animals by bilateral carotid artery occlusion (TIA) or middle cerebral artery occlusion (MCAO). Under anaesthesia, an incision will be made at the thoracic area. For TIA, both the left and right common carotid arteries will be clamped transiently for about 12 minutes. For MCAO, an intraluminal suture will be inserted from the external carotid artery to occlude the middle carotid artery transiently for about 2 hours. After surgery, the incision will be closed. The animals will be allowed to recover. Analgesics will be given for pain relief. The animals will then be divided into groups and treated orally with Chinese medicine formula or herbs, or distilled water as control. The behavioural performance of the animals will be evaluated by shuttle-box escape test. In the test, the animals will be presented with light and sound for about 10 seconds as conditioned stimuli, followed by minimal electric current for about 10 seconds as an unconditioned stimulus to trigger the animals to escape from one chamber to another. The conditions of the animals will be monitored during the experiment. Animals showing any signs of pain or distress will be euthanized before the end of the study. At the end of the experiment, the animals will be anaesthetised and cerebrospinal fluid will be aspirated for pharmacological studies. The animals will then be sacrificed by overdose of anaesthetics. Brain will be harvested for *in vitro* assays.

Place(s) where experiment(s) may be conducted

Laboratory Animal Services Centre, The Chinese University of Hong Kong

---

Conditions

1. Such experiment(s) may only be conducted for the following purposes-

   To investigate the neuroprotective effects of traditional Chinese medicine composed of Tianma (Rhizoma Gastrodiae Elatae) and Gouteng (Ramulus Uncariae cum Uncis) on TIA and MCAO rat models.

2. This licence is valid from 31 December 2010 to 30 December 2012

Dated 31 December 2010

DEPARTMENT OF HEALTH 衛生署 HONG KONG

Licensing Authority
