# Supplementary material for: Gastrodia and Uncaria (tianma gouteng) water extract exerts antioxidative and antiapoptotic effects against cerebral ischemia in vitro and in vivo
Source: Chin Med. 2016 May 31;11:27. doi: 10.1186/s13020-016-0097-6 (PMC4888490; doi:10.1186/s13020-016-0097-6)
Supplement: Supplementary file 5 — 10.1186/s13020-016-0097-6 Animal experimentation ethics committee approval protocol 3. [file 13020_2016_97_MOESM5_ESM.pdf]

AEEC Faculty of Medicine, CUHK

Application Guidelines | Membership & Terms of References | Download Form | Useful Informaiton | External Link | FAQs | Contact

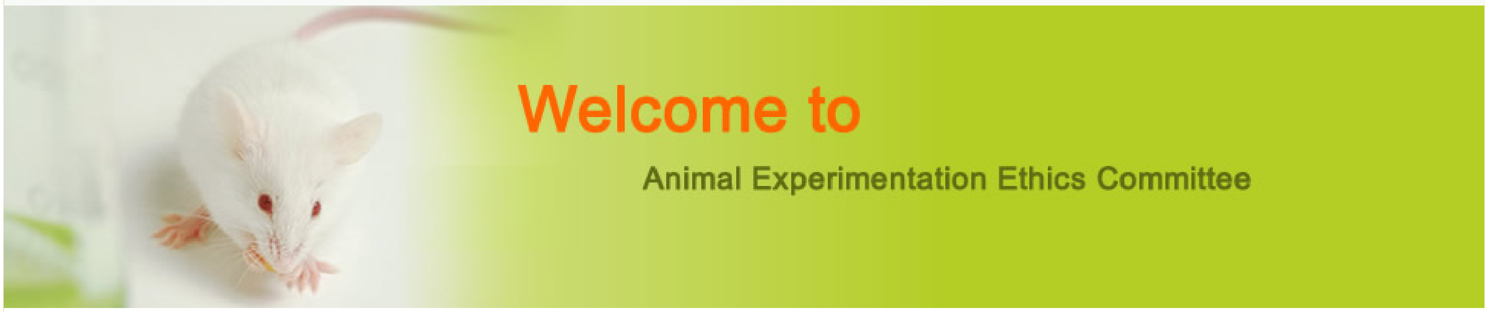

Membership and Terms of Reference

Membership

Chairman:	Professor LAN Hui Yao	Professor	Li Ka Shing Institute of Health Sciences, Faculty of Medicine
Members:	Professor CHEN Yangchao	Assistant Professor	School of Biomedical Sciences, Faculty of Medicine
	Professor CHEN Zhen Yu	Professor	School of Life Sciences, Faculty of Science
	Professor HUI Mamie	Associate Professor	Department of Microbiology, Faculty of Medicine
	Mr. HUI Lap Chung Stephen	Hostel Manager	Madam S.H. Ho Hostel for Medical Students
	Mr. LAM Shi Kai	Director	University Safety Office
	Dr. LEONG Yin-Ming Veronica	Veterinary Officer	Agriculture, Fisheries and Conservation Department, HKSAR Government
	Professor LI Gang	Professor	Department of Orthopaedics and Traumatology, Faculty of Medicine
	Reverend MILLER Stephen	Senior Chaplain	The Mission to Seafarers, The Mariners' Club
	Professor NG Chi Fai	Professor	Department of Surgery, Faculty of Medicine
	Dr. RIGGS Christopher M.	Head	Department of Veterinary Clinical Services, Equine Hospital, The Hong Kong Jockey Club
	Professor RUDD John A.	Professor	School of Biomedical Sciences, Faculty of Medicine
	Dr TANG Mei Kuen Florence	Lecturer	School of Biomedical Sciences, Faculty of Medicine
Ex-officio	Professor XIE Zuowei	Associate Dean (Research)	Faculty of Science
	Dr. ROWLANDS Dewi K.	Director	Laboratory Animal Services Centre, Faculty of Medicine
Secretary:	Ms. KONG Mui Kam Maria	Senior Technician	Faculty and Planning Office, Faculty of Medicine

Website: www.aeec.med.cuhk.edu.hk

Terms of Reference

1. To consider all ethical issues relating to the use of experimental animals for the purposes of research, teaching and other scientific investigations.
2. To ensure that all animal use procedures are conducted in such a way as to avoid any unnecessary usage, suffering and injury regarding the animals involved.
3. To review proposed animal use protocols and have the authority to approve, require modifications in (to secure approval), or disapprove such proposals.
4. To invite individuals with competence in special areas to assist in the review of complex issues which require expertise beyond or in addition to that available from among the committee.
5. To exercise authority delegated by the Research Committee when necessary to observe and monitor the implementation of the animal use protocols or to appoint a third party to observe and monitor the procedures.
6. To exercise authority delegated by the Research Committee when necessary to suspend or terminate approval of any animal use procedure that is not being conducted in accordance with generally accepted ethical requirements or that has been associated with unnecessary suffering or injury to the animal subject(s).

Any suspension or termination of approval will include a statement of the reasons for the committee's action. The investigator(s) and appropriate University official(s) shall be notified accordingly.

7. To report to the Research Committee annually the work of the committee, and report any special cases where appropriate.
