# Supplementary material for: Gastrodia and Uncaria (tianma gouteng) water extract exerts antioxidative and antiapoptotic effects against cerebral ischemia in vitro and in vivo
Source: Chin Med. 2016 May 31;11:27. doi: 10.1186/s13020-016-0097-6 (PMC4888490; doi:10.1186/s13020-016-0097-6)
Supplement: Supplementary file 3 — 10.1186/s13020-016-0097-6 Animal experimentation ethics committee approval protocol 1. [file 13020_2016_97_MOESM3_ESM.pdf]

FACULTY OF MEDICINE
THE CHINESE UNIVERSITY OF HONG KONG
香港中文大學
醫學院

TELEGRAM · SINOVERSITY
電報掛號 6331
TELEX · 專用電報訊號 50301 CUHK HX
FAX · 圖文傳真 (852) 2637 0979

PRINCE OF WALES HOSPITAL
SHATIN, N.T., HONG KONG
TEL : (852) 2632 2846

香港新界沙田
威爾斯親王醫院
電話：二六三二 二八四六

Your Reference :

Our Reference :

December 5, 2011

Professor LEUNG Wing Nang Albert
School of Chinese Medicine
Faculty of Science

Dear Professor Leung,

<u>Animal Experimentation Ethics Approval</u>

I am pleased to confirm that the Animal Experimentation Ethics Committee agrees to grant ethical approval for your research project entitled "The neuro-protective effect of Tianma-Gouteng formula on cerebral ischemia" (Ref No. 11/068/MIS).

Please also note that the approval is for the procedures described in your application and variation from these procedures requires an additional endorsement from the AEEC.

Thank you for your attention.

Yours sincerely,

Professor Christopher Haines
Chairman
Animal Experimentation Ethics Committee

cc Dr Anthony James, Laboratory Animal Services Centre
